# Supplementary material for: An electron transport complex required in the gut sensitizes Bacteroides to a pore-forming type VI secretion toxin
Source: Proc Natl Acad Sci U S A. 2025 Dec 9;122(50):e2523503122. doi: 10.1073/pnas.2523503122 (PMC12718326; doi:10.1073/pnas.2523503122)
Supplement: Supplementary file 1 — Appendix 01 (PDF) [file pnas.2523503122.sapp.pdf]

PNAS

Supporting Information for:

An electron transport complex required in the gut sensitizes Bacteroides to a pore-forming type VI secretion toxin

Hannah K. Ratner[a], Brandon D. Duong[a], Pengrui Miao[a], Savannah K. Bertolli[a], Beth A. Shen[a], Uma Mitchell[a], Larry A Gallagher[a], Matthew C. Radey[a], S Brook Peterson[a,d], and Joseph D. Mougous[a,b,c,d*]

[a]Department of Microbiology, University of Washington, Seattle, WA, 98109
[b]Howard Hughes Medical Institute, University of Washington, Seattle, WA, 98109
[c]Microbial Interactions and Microbiome Center, University of Washington, Seattle, WA, 98195
[d]Current address: Department of Microbial Pathogenesis and Microbial Sciences Institute, Yale University School of Medicine, New Haven, CT 06536

* To whom correspondence should be addressed:
Email – joseph.mougous@yale.edu

This PDF file includes:

Figures S1 to S4
Legends for Datasets S1 to S4

Other supporting materials for this manuscript include the following:

Datasets S1 to S4

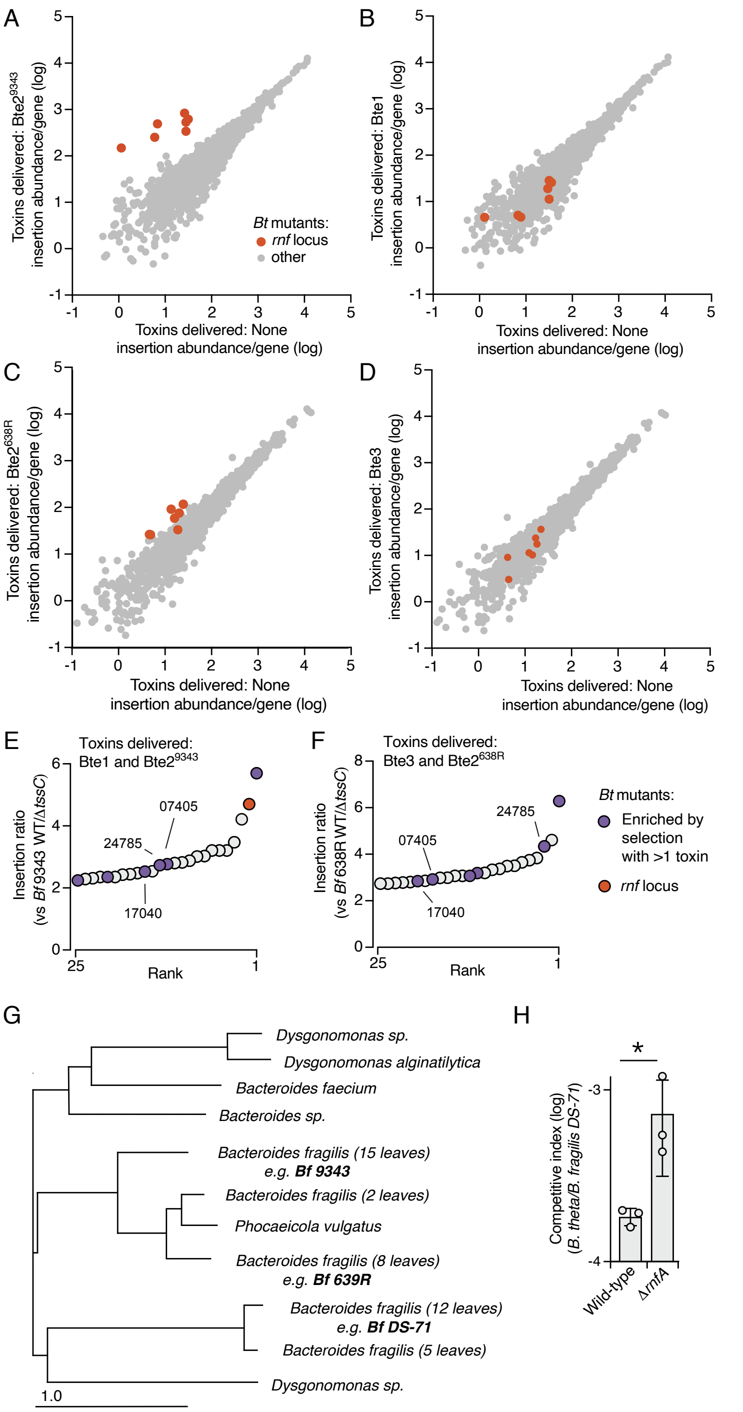

Fig S1. Transposon mutant screening and growth competition assays to identify *Bacteroides* T6SS intoxication susceptibility determinants. A-D) Transposon insertion abundance per *Bt* gene during antagonism with *Bf* strains delivering the indicated individual T6SS toxins (y-axis) or *Bf*Δ*tssC* (inactivated T6SS, x-axis). **E-F)** Rank fold enrichment of *B. theta* (*Bt*) transposon insertion mutants following growth in competition with the indicate WT *B. fragilis* strains. *Bt* mutants specifically mentioned in the text are highlighted BT_RS24785, AAA-family ATPase; BT_RS07405, hypothetical; BT_RS17040, predicted glycosyltransferase. **G)** Phylogeny of Bte2 family proteins in *Bacteroidales*. Bte2 orthologs tested for Rnf-dependence via

competition growth assays are indicated in bold. **H)** Outcome of growth competition assays between *Bt* (wild-type or Δ*rnfA*) and *B. fragilis* DS-71. Data represent mean ± SD (*p<0.05, unpaired one-tailed Student's *t*-test, n=3).

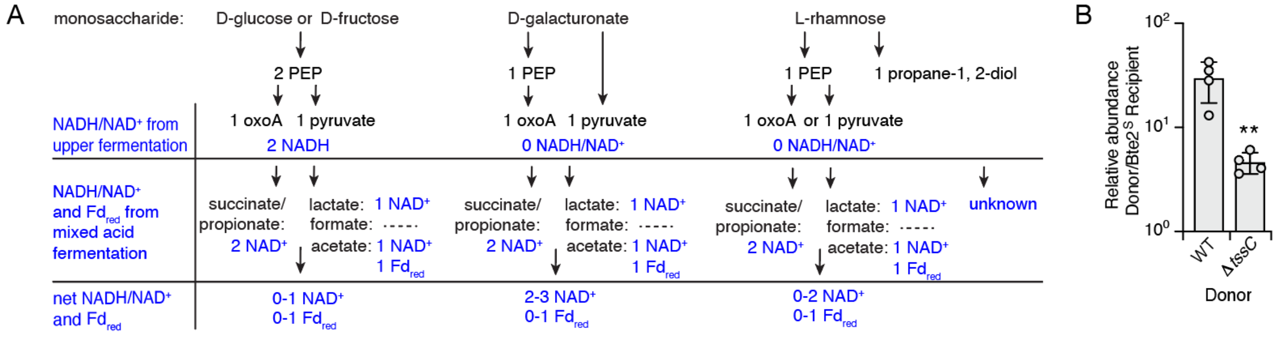

Fig. S2. *B. theta* cofactor utilization varies during growth on different monosaccharides and Bte2 improves *Bf* fitness in vivo. A) Predicted NADH/NAD$^+$ and $Fd_{red/ox}$ utilization in *Bt* as determined by Kegg pathway analysis during growth on the indicated monosaccharides. B) Abundance of *Bf* (*Bf* 638R WT or Δ*tssC*) relative to a Bte2-susceptible variant (*Bf* 638R Δ*btei2*) in cecal contents 10 days after pairwise gavage into gnotobiotic mice. Data represent mean ± SD (**p<0.01, unpaired two-tailed Student's *t*-test, n=4).

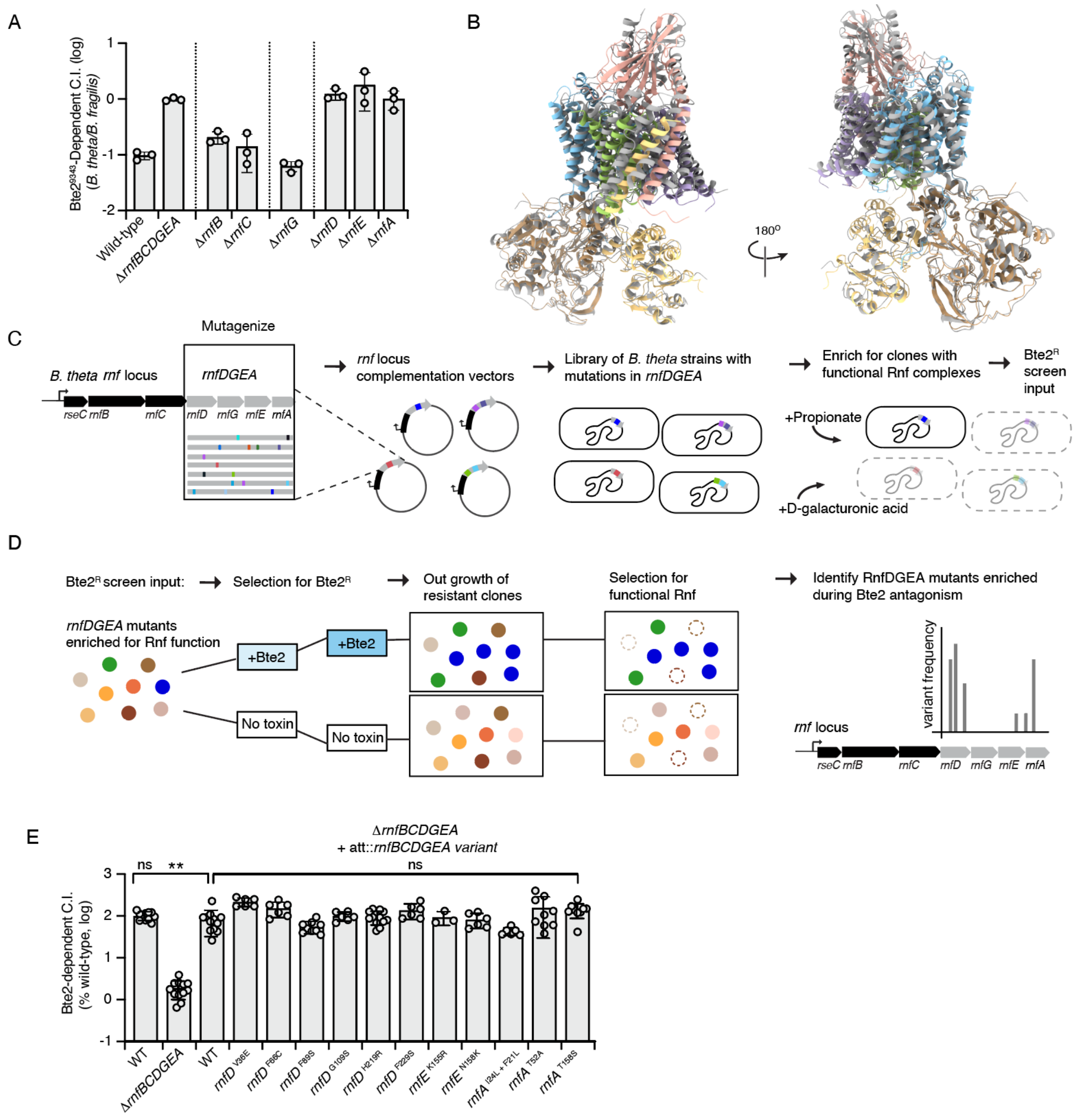

Fig. 3. RnfDEA are not permissive to mutations that resist Bte2 and maintain Rnf function. A) Competitiveness of *Bt rnf* locus variants during growth competition assays with *Bf* delivering Bte2^{9343} relative to in similar assays with *Bf* delivering no toxins. **B)** Alphafold3 multimer-generated model of the *Bt* Rnf complex (subunits colored according to Fig. 2A) overlayed with the solved structure of the Rnf complex from *Clostridium tetanomorphum* (grey, PDB: 7zc6 (46)). **C,D)** Schematic depicting construction of a library of *Bt* with randomly mutagenized *rnfDGEA* subunits and initial selection for Rnf complex function (C) and design for the screen to identify Bte2-resistant variants in the library of *Bt* with mutagenized *rnfDGEA* (D). **E)** Relative competitiveness the indicated reconstructed variants of *Bt rnfDEA* during growth competition assays with *Bf* delivering Bte2^{638R} relative to competition with *Bf* delivering no toxins (shown as % WT *Bt* fitness) (n=3-12). Variants represent those selected by Bte2 exposure during our genetic screen. Data represent mean ± SD (**p<0.05, unpaired two-tailed Student's *t*-test, n=3).

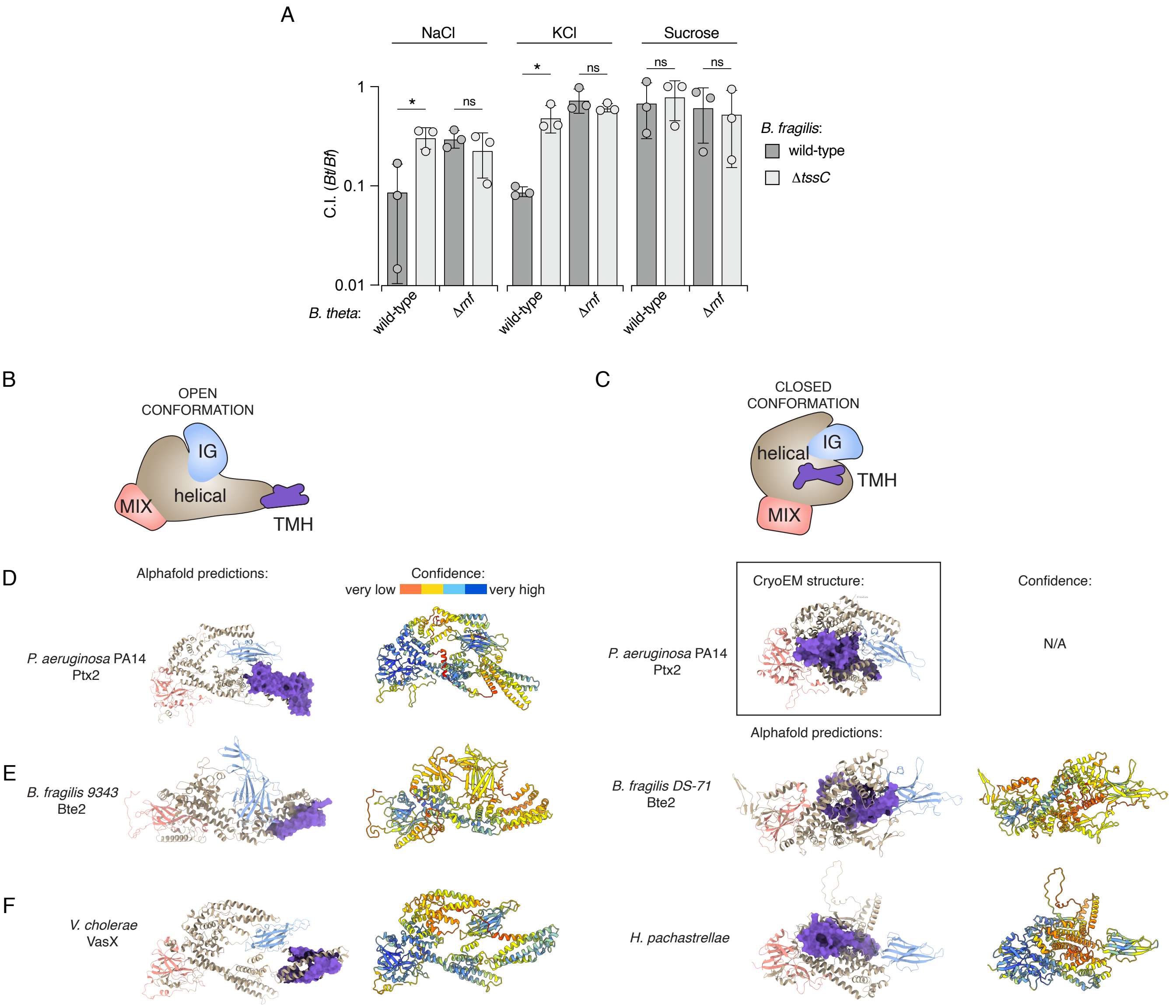

Fig S4. Structure modeling of VasX-family proteins predicts variable conformations for the helical domain. A) Competitiveness of *Bt* (wild-type or Δ*rnfBCDGEA*) during growth competition assays with *Bf* delivering Bte2^{638R} or no toxins (D*tssC*), on media supplemented with different osmoprotectants. Data represent mean ± SD (*p<0.05, 1-way ANOVA with Sidak's multiple comparisons test). **B, C**) Schematics depicting a generalized VasX-family protein in the open (**B**) or closed (**C**) conformations. **D**) Alphafold3 prediction (left) and CryoEM structure of Ptx2 from *P. aeruginosa* (right). Model predictions are colored by domains (left, colors correspond to A and B) or model confidence (right). **E, F**) Alphafold3 models of representative VasX family proteins, colored by domains and model confidence. Examples were selected to indicate related proteins from *Bacteroides fragilis* (**E)** or g-Proteobacteria (**F**) that have different predicted conformations.

Dataset S1 (separate file). Normalized transposon insertion frequencies per gene of *B. thetaiotamicron.* Insertion frequencies following growth of a barcoded transposon insertion library in competition with the indicated competitors, related to Figure 1 and Figure S1.

Dataset S2 (separate file). RnfDGEA variant frequencies before and after Bte2-selection. The starting pool of *B. theta* clones with randomly mutagenized RnfDGEA subunits was plated on Rnf-selective media for functional Rnf complexes. The variant frequency indicates the % frequency of each mutation in each sample. The control is the average of the variant frequencies from the samples that were not exposed to Bfe2-intoxication but were selected for functional Rnf (the input library pool after selection for Rnf function and library pool competed with *B. fragilis* Δ*tssC* followed by a second selection for Rnf function). The screen output is input library after two rounds of selection with Bfe2 and a second selection for functional Rnf complexes. Related to Figure 3.

Dataset S3 (separate file). Analysis of representative Bte2-encoding species. Genomes analyzed for the Rnf locus and immunity to Bte2, related to Figure 4E.

Dataset S4 (separate file). Strains, plasmids and primers used in this study.
